# Calibration and Modeling of the Semmes–Weinstein Monofilament for Diabetic Foot Management

**DOI:** 10.3390/bioengineering11090886

**Published:** 2024-08-31

**Authors:** Pedro Castro-Martins, Luís Pinto-Coelho, Raul D. S. G. Campilho

**Affiliations:** 1 CIETI, ISEP, Polytechnic of Porto, 4249-015 Porto, Portugal; pmdcm@isep.ipp.pt; 2 Faculty of Engineering, University of Porto, 4200-465 Porto, Portugal; 3 INESC TEC—Institute for Systems and Computer Engineering Technology and Science, 4200-465 Porto, Portugal; 4 CIDEM, ISEP, Polytechnic of Porto, 4249-015 Porto, Portugal; rds@isep.ipp.pt; 5 INEGI, Institute of Science and Innovation in Mechanical and Industrial Engineering, 4200-465 Porto, Portugal

**Keywords:** diabetic foot, monofilament, metrological verification, buckling force

## Abstract

Diabetic foot is a serious complication that poses significant risks for diabetic patients. The resulting reduction in protective sensitivity in the plantar region requires early detection to prevent ulceration and ultimately amputation. The primary method employed for evaluating this sensitivity loss is the 10 gf Semmes–Weinstein monofilament test, commonly used as a first-line procedure. However, the lack of calibration in existing devices often introduces decision errors due to unreliable feedback. In this article, the mechanical behavior of a monofilament was analytically modeled, seeking to promote awareness of the impact of different factors on clinical decisions. Furthermore, a new device for the automation of the metrological evaluation of the monofilament is described. Specific testing methodologies, used for the proposed equipment, are also described, creating a solid base for the establishment of future calibration guidelines. The obtained results showed that the tested monofilaments had a very high error compared to the 10 gf declared by the manufacturers. To improve the precision and reliability of assessing the sensitivity loss, the frequent metrological calibration of the monofilament is crucial. The integration of automated verification, simulation capabilities, and precise measurements shows great promise for diabetic patients, reducing the likelihood of adverse outcomes.

## 1. Introduction

**Figure 1:**
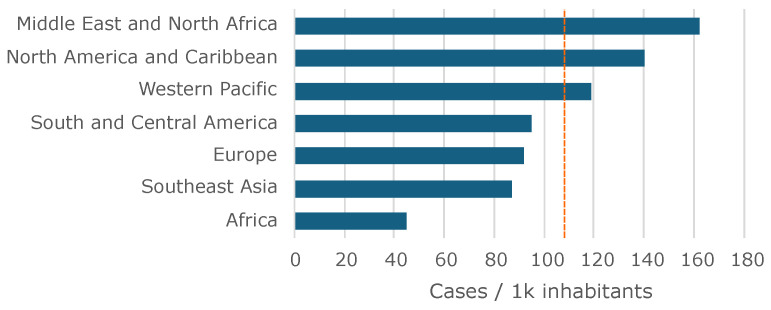
Worldwide prevalence of diabetes per region. Average is represented by the vertical dashed line.

Diabetes, a chronic disease, is witnessing a great surge in global incidence, particularly in most developed countries. Every year, around 1.5 million deaths worldwide are directly attributed to diabetes [1]. Middle East and North Africa areas are particularly affected, as can be observed in Figure 1 [2]. The disease can present several complications, and when coexisting with other comorbidities, serious problems arise for the patient, as is the case of the diabetic foot. This condition manifests as lower limb complications, predominantly in the plantar region, whose evolution can have devastating effects and with a high probability of compromising part of the affected limb [3]. Although it is possible to minimize the occurrence of ulcers by carefully monitoring the feet and using appropriate footwear [4,5], as a preventive measure, clinical tests must be periodically conducted to assess the loss of skin sensitivity to pressure on the foot, aiming to preclude imminent injuries. The 10 gf Semmes–Weinstein monofilament (SWM) test is the most used first-line instrument in the assessment of the loss of protective sensitivity in the diabetic foot, with well-established international recommendations for its widespread use [3,6].

**Figure 2:**
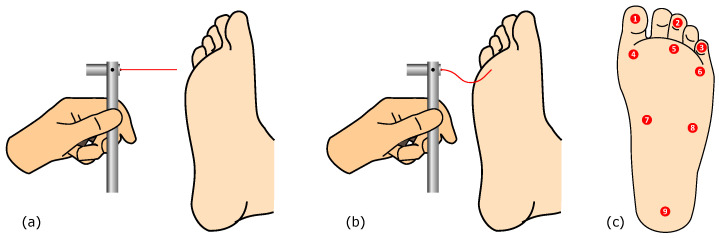
Plantar sensitivity assessment using the monofilament, showing (**a**) the approaching stage; (**b**) the contact stage, with visible buckling; and (**c**) the related evaluation points, for the left foot.

The SWM is a widely used diagnostic tool designed to assess and quantify sensory loss, particularly in individuals with conditions such as diabetes, peripheral neuropathy, or Hansen’s disease (leprosy). The tool consists of a set of monofilaments of varying thicknesses, made of nylon (or analogous flexible plastic material, sometimes unspecified), each estimated to bend when a specific amount of force is applied. During a sensory test, a clinician applies the monofilament perpendicular to the skin until it bends, holding it in place for about one to two seconds. This procedure is repeated at several locations, on the foot or other relevant areas of the body, to assess sensory perception. This design allows healthcare providers to evaluate a patient’s ability to perceive light touch at different thresholds, which is crucial in detecting and monitoring sensory deficits. The test helps to determine the smallest force a patient can feel, which in turn classifies the degree of sensory loss. For example, a person who can only perceive thicker filaments, which require more force to bend, has greater sensory impairment than someone who can detect thinner filaments [7]. In the particular case of the diabetic foot, a single 10 gf monofilament is used, and the test locations are on the plantar area, as depicted in Figure 2 (though some additional locations on the foot dorsum can be covered as part of the screening process).

Traditionally, this assessment is conducted manually by experienced healthcare professionals, although automation through collaborative robotic systems is also emerging as a viable alternative [8,9]. Despite the wide usage of this test, its qualitative nature lacks the capability to provide numerical data, posing a challenge for objective comparison among screening tests. Additionally, given the lack of accuracy of these devices, considering the same patient, it may be possible to obtain different plantar sensitivity responses when using two different monofilaments [10].

Moreover, the inherent limitations of the SWM test extend to its uncertainty concerning measurement errors, hindering its utility as a decision-making support tool. Despite the literature advocating for quantitative assessments of sensory loss severity in diabetic foot cases, the SWM test fails to provide definitive insights. Consequently, some scholars express reservations regarding the reliability of the force applied by the SWM on the patient’s skin during sensitivity screening tests [11,12,13,14]. In fact, in a systematic review with meta-analysis [10], it is reported that monofilament tests have limited sensitivity for screening diabetic peripheral neuropathy and that their clinical use in the evaluation of diabetic peripheral neuropathy could not be encouraged. This reinforces the present study indicating the use of the monofilament can be encouraged as long as the operation characteristics of the monofilament are known and monitored, as is the case with other medical devices.

Concerning the monofilament operating principle, one issue to consider is that the compressive force decreases with the number of successive uses, resulting in a significant change in its initial elastic characteristics. This phenomenon introduces potential inaccuracies in subsequent assessments, which is critically reinforced by the uncertainty surrounding the threshold at which such errors become significant [12]. Furthermore, environmental factors such as temperature and relative humidity can exert considerable influence on the compression force, dimensions, and the subsequent deformation of the SWM. Laboratory tests conducted under varying environmental conditions, including controlled temperature and humidity levels of up to 37°C and 80%, demonstrated a progressive decrease in the force exerted by the monofilament by approximately 40% [15]. If a nylon product absorbs moisture, dimensional changes will occur with increasing moisture content [16]. This effect is directly proportional to the exposed surface area, as occurs in monofilaments. However, moisture-related variations are small in controlled environments such as most healthcare facilities, making this a negligible effect for the current study.

The evaluation of the SWM performance, as a clinical tool, is still rather unexplored, despite its impact on medical decisions. Nevertheless, this technique is widely used due to its simplicity, practicality, and low cost. As a routine screening device, it is used to select diabetics who still have protective sensitivity from those who do not [3,7,17], guiding the definition of the follow-up processes. However, there are guidelines that warn healthcare professionals about the possible loss of flexion strength of the monofilament over time and that this deterioration can result in inconsistent force application, deviating from the manufacturer’s specified standards [17]. Consequently, healthcare providers are urged to have access to equipment that enables them to monitor whether a given monofilament maintains the required force levels for diabetic foot screening (and also for other clinical applications where this instrument is used).

The main contributions of this article are highlighted in the following points:

- An analytical model for the mechanical behavior of the SWM is presented. This helps to understand the individual impact of each variable on the behavior of the monofilament during its use. On the other hand, it allows for establishing operational ranges for the parameters of the usage environment in order to maintain the measurement characteristics.
- A new metrological testing device is described, expanding a previous version [18], which aims to evaluate the behavior of a given monofilament. This equipment’s main functionalities are quantifying the deviation of an SWM in relation to the 10 gf standard, and carrying out fatigue tests. There is also the possibility of carrying out other types of studies.
- A testing methodology for monofilaments is also presented, establishing a possible reference procedure for the creation of international guidelines regarding their metrological behavior.

The rest of this paper is organized as follows: The modeling of the monofilament behavior under plantar sensitivity test conditions is first presented. This helps to understand the critical parameters to observe during usage and during the devices’ calibration. Then, a new device is thoroughly described, designed to evaluate the SWM in terms of its real compression force and, with this, to provide information to healthcare professionals about the force range for a monofilament. Several tests are then performed with a sample group of monofilaments. The obtained results are presented and widely discussed. Finally, the main aspects of the study are highlighted as well as some prospects for future work.

## 2. An Analytical Model of the Semmes–Weinstein Monofilament

When performing the diabetic foot plantar sensitivity test using a monofilament, it is recommended to use monofilaments with specific characteristics to ensure consistent and standardized results. The SWM test is commonly used for this purpose, and it is commercially available in a set of distinct target force values with specific characteristics. These devices consist of nylon filaments that can have different diameters and lengths to apply a specific force to the skin when they are pressed and buckling. Usually referred to in grams by the medical community, the most commonly used target levels in the SWM test include 0.07 g, 0.16 g, 0.4 g, 0.6 g, 1 g, 2 g, 4 g, 10 g, and 300 g [19,20]. These target levels allow for the evaluation of different levels of tactile sensitivity, with a 10 g monofilament being recommended for the evaluation of the diabetic foot [21].

Each monofilament is usually labeled with a number (during manufacturing) representing the force required for buckling to occur. These numbers range from 1.65 to 6.65 (evaluator size, a dimensionless scale) and indicate different standardized pressure values to be applied; the higher the number, the greater the force required for the buckling of the monofilament and therefore the greater the pressure exerted on the skin. For example, the evaluator size of 5.07 concerns a 10 g monofilament and, as such, the manufacturer states that a force of 10 gf is required for its buckling [19]. If this monofilament is applied to a specific point on the foot, and the patient cannot feel the pressure, this may indicate a loss of sensitivity in that region [21].

### 2.1. Materials

The monofilament is generally made of nylon, which is a generic designation for a family of synthetic polymers composed of polyamides. To specifically understand how the material behaves mechanically during operation and use, it is necessary to have particular knowledge about it.

Concerning the material’s properties, the most relevant to consider is Young’s Modulus. This property represents the stiffness of the nylon fiber, and it affects its resistance to buckling, with a higher Young’s modulus indicating greater stiffness, hence resisting buckling. The Yield stress and Poisson’s ratio can also be considered. These represent the ability to withstand deformation before experiencing permanent damage or failure and a measure of the material’s lateral contraction when subjected to axial loading, respectively.

Extruded Nylon 6.6 (extruded polyamide 6.6) is the most common type, which is known for its high strength, stiffness, and excellent resistance to wear. However, it can be affected by moisture absorption, causing slight changes in its mechanical properties, including Young’s modulus. Nylon 6, an alternative also widely used, has good mechanical strength, toughness, and abrasion resistance, being generally less affected by humidity compared to other types of nylon. Humidity absorption by nylon fibers can cause changes in the material’s ability to resist compression or deflection. The relationship between humidity and Young’s modulus can be described using the following empirical equation [22]:

(1) $$E\left(H\right)=E_{0}\cdot \left(1-\beta \cdot (H-H_{0})\right)$$

where E(H) is Young’s modulus at humidity *H*, $E_{0}$ is the reference Young’s modulus at a reference humidity $H_{0}$, and β is the humidity coefficient. The humidity coefficient (β) represents the rate at which Young’s modulus changes with humidity and is typically provided by the material manufacturer, varying for different types of nylon. Humidity acts as a plasticizer in nylon and therefore reduces strength and stiffness properties but increases elongation and toughness. In general, as humidity content rises, significant increases occur in impact strength and other energy-absorbing characteristics of the material.

The Young’s modulus of a material usually decreases as temperature increases. This inverse relationship can be modeled using the following equation:

(2) $$E\left(T\right)=E_{0}\cdot \left(1-\alpha \cdot (T-T_{0})\right)$$

where E(T) is Young’s modulus at temperature *T*, $E_{0}$ is the reference Young’s modulus at a reference temperature $T_{0}$, and α is the temperature coefficient of the material. The temperature coefficient (α) represents the rate at which Young’s modulus changes with temperature. Like humidity, it can vary for different types of nylon and is typically provided by the material manufacturer.

Besides material factors, geometry also plays a role. Length and diameter, or more specifically the length-to-diameter ratio of the fiber, is a critical geometric factor affecting buckling behavior. Longer fibers or fibers with smaller diameters are more prone to buckling. In addition, imperfections such as bends, eccentricities, or deviations from a perfect straight form, can amplify the tendency for buckling. Imperfections act as localized stress concentrations and can initiate buckling at lower loads. Imperfections can be a result of the manufacturing process but also a consequence of monofilament misuse (for example, when objects inadvertently cause permanent plastic deformations due to placing the monofilament device in a clothing pocket exposed to keys, a pen, or others). The boundary conditions imposed on the fiber ends can also significantly influence buckling [23]. In most cases, a fixed at one edge and pinned at the other (fixed–pinned) coupling is considered, but different skin stiffness can alter this behavior, leading to variations in buckling modes and critical buckling loads.

Finally, loading conditions must also be taken into account. The magnitude and direction of the applied compressive load directly influence the buckling behavior, with higher compressive loads increasing the risk of buckling. The rate at which the load is applied can also affect buckling behavior. Rapid loading or dynamic loading conditions may lead to different buckling responses compared to static loading [24].

It is important to note that the abovementioned variables are interconnected, and their combined effects need to be considered in evaluating column buckling in a nylon fiber. Analytical methods, such as Euler’s buckling theory or finite element analysis, can be employed to assess the influence of these variables and predict both the critical buckling load and mode of failure for a specific nylon fiber column. Experimental testing can also provide valuable insights into the buckling behavior by considering these variables in a real-world scenario. In Table 1 a summary of which material characteristics can affect the monofilament measurement performance is presented.

### 2.2. Buckling

In practice, it is observed that 10 gf monofilaments are commercially available with a wide range of diameters and small variations in length. Most of the time, no information is given about the material from which it is produced. These characteristics can strongly impact the behavior of the monofilament when in operation, as will be shown in the following sections of this study.

The buckling equation [25], also known as Euler’s buckling equation, allows for the estimation of the force that leads to the initial buckling. It is given as follows:

(3) $$F_{c}=\frac{\pi^{2}\cdot E\cdot I}{L_{e}^{2}}$$

where $F_{c}$ is the critical buckling force, *E* is the Young’s modulus of the material, *I* is the 2nd moment of inertia of the cross-sectional area, and $L_{e}$ is the effective length of the filament. The effective length is calculated as $L_{e}=k\cdot L$, where *k* is defined taking into account the boundary conditions, and *L* is the real length of the monofilament. The end near the handle of the monofilament will always be clamped, while for the free end, two possibilities can be considered. The first case will correspond to the most common situation in which the point of contact with the skin has no slippage and which, due to the small diameter of the filament, can be considered a hinged–pinned connection. When the skin surface at the contact point does not guarantee sufficient friction (due to oil, dryness, sweat dirt, or other factors), then there will be a hinged connection with sliding. For the clamped–pinned case, a theoretical k=0.7 was defined, and for the clamped–(pinned and sliding) case, k=2.0 was the reference (clamped means rotation, and translation-fixed, pinned, and sliding means rotation and translation-free) [26].

To calculate the second moment of inertia (*I*) for a circular cross-sectional area, the following formula is used:

(4) $$I=\frac{\pi \cdot d^{4}}{64}$$

where *d* is the diameter of the filament.

Hence, defining the monofilament material, length, and diameter allows us to estimate its critical buckling force. On the other hand, if the critical force is known, as well as the geometrical dimensions, then Young’s modulus for an unknown material can be estimated.

### 2.3. Deflection

During the evaluation of plantar sensitivity, the monofilament is positioned horizontally to move perpendicularly toward the patient’s foot. In this position, the nylon filament behaves like a cantilever beam subjected to distributed load, corresponding to its own weight, that will cause the deflection of the free extremity.

When a beam is not flexed or deflected, the applied load is mainly an axial load, meaning a force that acts along the longitudinal axis of the beam. The material of the beam is more efficient in supporting this type of load, as it is designed to resist compression and tension forces along its length. In this case, the load is transmitted directly to the supporting base, making the structure more stable and resistant.

On the other hand, when a beam is bent or flexed, it encounters extra forces known as bending moment and shear. This bending action causes the particles within the beam’s material to move relative to each other, creating internal stresses. These internal stresses, if excessive or repeated, have the potential to weaken the structure. The bending moment is a specific type of force during this bending process, which includes both a bending force (theforce bending the beam) and torque (a twisting force). Over time, these combined forces from the bending moment can lead to deformation and, ultimately, material failure. In simpler terms, when a beam bends, it experiences forces that can strain its material and gradually compromise its strength and shape. Additionally, shear, which is a cutting force perpendicular to the axis of the beam, can also affect the load-carrying capacity of the structure.

In this context, let us estimate the deflexion of the monofilament due to its weight. Considering a linear elastic deformation and a small deflection (<1/10 of span), the displacement of the monofilament-free extremity can be calculated from the general elastic equation [26], which is as follows:

(5) $$\frac{d^{2}y\left(x\right)}{dx^{2}}=\frac{M}{E\cdot I}$$

where the *x* axis is defined along the length *L* of the monofilament, *M* is the bending momentum, *E* is Young’s modulus, and *I* is the moment of inertia (Equation (4)). The monofilament weight is given as follows:

(6) w=V·ρ·g

where *V* is the volume of the monofilament, ρ the material’s density, and *g* is the gravity. In the present case, the load is distributed along the monofilament, and it can be expressed as q=w/L. In these conditions, the monofilament elastic deflection in the free extremity y(L)=δ can be written as follows:

(7) $$\delta =\frac{w\cdot L^{4}}{8\cdot E\cdot I}$$

## 3. Monofilament Calibration Equipment

Medical device calibration holds significant importance within the healthcare industry for several compelling reasons. Firstly, calibration ensures the provision of accurate and reliable measurements by medical devices. This precision is paramount for diagnosing medical conditions, determining suitable treatment options, and effectively monitoring patient health. By establishing consistency and accuracy in readings, calibration contributes to the overall quality of healthcare delivery. Secondly, the major concern of patient safety is intricately linked to medical device calibration. An incorrectly calibrated device can lead to erroneous readings, potentially resulting in misdiagnosis, inappropriate treatment, or patient harm. Regular calibration practices serve as a crucial mechanism to identify and rectify any deviations or inaccuracies, thus minimizing the risk of adverse events and fostering patient well-being [27]. Moreover, compliance with regulatory standards and guidelines is of utmost importance in the healthcare domain. Calibration is an indispensable component in meeting these requirements, as regulatory bodies, such as the Food and Drug Administration (FDA), often mandate regular calibration of medical devices to ensure both patient safety and device performance. Failure to comply with these regulations can result in severe consequences such as penalties, legal ramifications, and reputational damage for healthcare institutions [28].

Calibration serves as an integral part of quality control programs, ensuring that medical devices consistently adhere to established standards and specifications. By systematically calibrating devices at appropriate intervals, healthcare organizations can promptly identify and rectify any deviations or discrepancies in device performance. This proactive approach significantly contributes to maintaining the overall quality and reliability of diagnostic and therapeutic procedures, thereby fortifying the foundations of healthcare provision. In addition, calibration plays a pivotal role in preserving data integrity within healthcare settings. Numerous medical devices are seamlessly integrated with electronic health record (EHR) systems or employed for clinical research purposes. Calibration practices ensure the accuracy and reliability of the data generated by these devices, thereby bolstering the credibility of data analysis, research outcomes, and evidence-based decision-making within the medical community [29].

Another notable aspect lies in the extended lifespan and enhanced performance of medical devices due to regular calibration. By effectively detecting and rectifying any inaccuracies or signs of wear and tear, calibration mitigates the risk of premature device failures. Consequently, this practice reduces equipment downtime, minimizes repair costs, and optimizes operational efficiency within healthcare facilities, facilitating seamless healthcare delivery [29,30].

Lastly, calibration contributes to traceability and standardization within the healthcare sector. The process involves comparing measurements obtained from a medical device against a traceable reference standard, establishing a chain of traceability that ensures consistent measurements across different devices and locations. This standardization facilitates effective communication, collaboration, and data sharing among healthcare providers and institutions, promoting a cohesive healthcare ecosystem [30].

In conclusion, medical device calibration holds a pivotal position in healthcare, warranting attention due to its role in enabling accurate measurements, ensuring patient safety, maintaining regulatory compliance, supporting quality control initiatives, preserving data integrity, optimizing device longevity, and fostering traceability and standardization. As such, calibration practices are instrumental in delivering effective healthcare and promoting favorable patient outcomes [31].

### 3.1. Equipment Architecture

**Figure 3:**
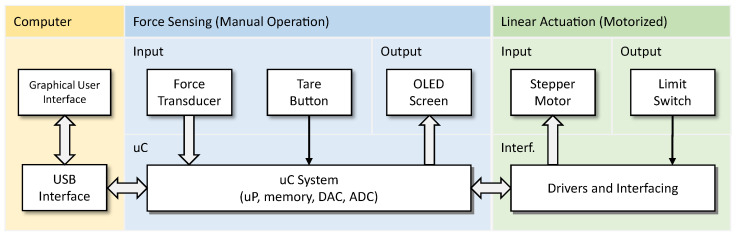
Main equipment components and their interaction: uC—microcontroller, uP—microprocessor, DAC—digital to analog converter, ADC—analog to digital converter, Interf.—interface.

The proposed device consists of two elements that can work together or independently. First, it is a force sensor system that can measure and display the applied force. The other is an accurate linear displacement system for simulating various SWM application scenarios. The various components of the developed device and their interactions are shown in the diagram in Figure 3. The equipment should be interfaced with a computer to allow for better equipment operation, for the observation of evaluation charts, data persistence, and statistics, among other functionalities. During development, preference was given to standard components already tested and available on the market. Where this was not possible, specific parts were designed from scratch and designed and simulated using 3D modeling software (Solidworks 2023) to ensure all project requirements and desired properties were met. Prototypes were then printed with 3D printing technology using PLA (a type of organic biodegradable thermoplastic polymer), tested, and improved when necessary. The produced parts proved to have good mechanical resistance and operational reliability in the desired operation conditions, thus exhibiting excellent performance for their intended purpose.

### 3.2. Force Sensing System

**Figure 4:**
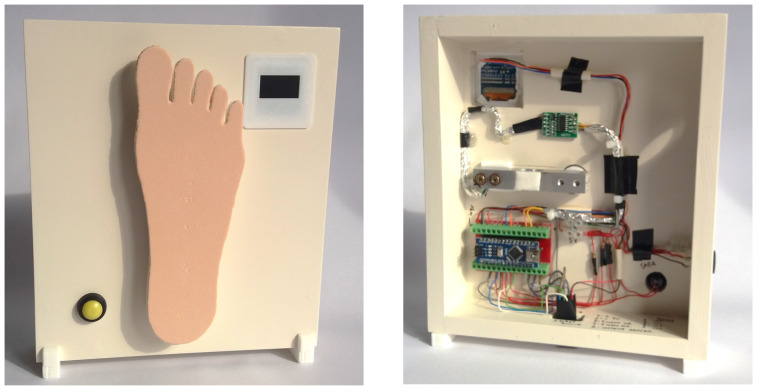
Force sensing system and its measurement platform are shaped similarly to the plantar region of the foot: external view of the module for manual measurement (**left**) and inner hardware components (**right**).

This is a portable element and can be operated alone to carry out measurements with manual application of the monofilament and take measurements, thereby sensing the value of the applied force. However, it can also be coupled with the precision linear displacement system (PLDS) (described next) and work together as a single mechanism in automatic mode. The force sensing system (FSS) (see Figure 4), in summary, is equipped with an OLED screen, a force transducer, a button to carry out the tare measurement, a connector for power input, and a USB Mini-B input for communication with a computer. Finally, an electronic controller (composed of a microprocessor, memory, and digital converters), based on the IC ATmega328, is responsible for carrying out all the programmed operations and executing the commands that will be sent through a computer application. When in automatic mode, the computer fully controls the equipment, i.e., when the FSS is coupled with the PLDS. The fundamental element consists of a force measurement platform, shaped similarly to the morphology of the plantar region of a human foot, which, in turn, is coupled to the force transducer. The surface of the platform has an ethylene–vinyl acetate coating with a texture and hardness chosen to be similar to what is observed with real biological tissues. It will be on this platform that the monofilament under evaluation will apply its strength, in a movement similar to the technique used in the sensitivity evaluation of the diabetic foot. Through this measurement process, it is be possible to obtain the respective inherent value of the buckling force applied by the SWM.

### 3.3. Precision Linear Displacement System

**Figure 5:**
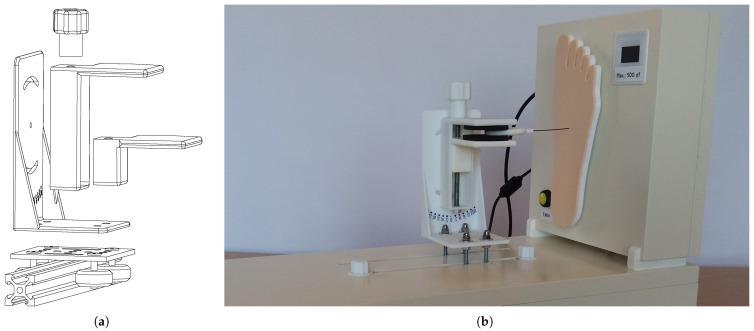
Proposed equipment, with a detailed view of the precision linear displacement system integrated with the force sensing system platform: (**a**) CAD drawing of the PLDS system with expanded components; (**b**) photographic image of the implementation of the PLDS and FSS.

This PLDS mechanism is responsible for the motorized application of the SWM. Therefore, for this mechanism to operate, it has to be coupled to the force sensing component, since, in turn, this is what contains all the control electronics and computer interfacing (through a dedicated application) and is responsible for measuring the buckling force of the monofilaments that will be subject to metrological verification. The PLDS element is composed of a stepper motor, a v-shaped sliding guide, a trolley with bearings, and a fixation support for the SWM. This set of components will carry out the movements to apply the strength of the monofilaments, simulating the technique used by health professionals on the diabetic foot assessment. In Figure 5, the full equipment can be observed in detail. The PLDS, depicted in a lower position, has visible slots for the rods connecting the visible tray with the motorized mechanism, hidden below. The top clamp, mounted on the tray, is used to robustly fixate the SWM, as shown in the figure. It allows us to adjust pitch and yaw incidence angles, creating the possibility for different testing conditions. Finally, in the rightmost area, the FSS platform (already described) can be observed, which in this case is operating as an integrated system with the PLDS.

The hidden stepper motor is responsible for moving a reinforced rubber belt, connected to a guided chariot. With this setup, it is capable of performing precise linear movements. It basically consists of the movement of the visible tray that follows with the SWM attached and simulates the technique of applying the monofilament to the patient’s foot. The monofilament is then moved and comes into contact with the measurement platform, where the applied force is measured, as this platform is coupled to the force transducer. These displacement movements of the monofilament fixation support are provided by the gantry and v-shaped sliding guide. The motion is supported by a transmission belt that is engaged in a pulley coupled to the stepper motor shaft, ensuring the desired precision. The displacement is electronically controlled and can be performed in linear and continuous movements, forwards and backwards, with end-of-course control or the number of steps taken.

### 3.4. Equipment Technical Specifications

Since the proposed equipment is intended to serve as a measurement system, it is important to consider the inherent accuracy and precision characteristics of force transducers, the components that convert physical quantities into corresponding electrical signals. To ensure that the equipment transmits quality estimates of the applied forces, it was subjected to a calibration process using a compression machine (Multitest 10-i, MecMesin, West Sussex, UK) with a load cell. This compression machine is approved to carry out calibration tests, and its main characteristics are detailed in Table 2. This step allowed for adjustments to the proposed equipment’s measurement algorithm to ensure precise and accurate measurements in a range of 0.1 gf to 500 gf.

Although the developed equipment has the capacity to carry out accurate measurements in a wider measurement range, for safety reasons, the force transducer was limited to 500 gf of maximum admissible load, which is within the expected SWM test values. This step ensures the integrity of the equipment and makes it possible to adapt it to other assessments with monofilaments of a caliber greater than 10 gf (there are calibers of up to 300 gf on the market, more commonly used to assess deep sensitivity). If 500 gf is exceeded, all system operation is aborted, and an error message appears on the screen, instructing the user to remove the load and reset the equipment. The internal resolution of the sensing stage is high (the integrated analog-to-digital conversion offers a 10-bit resolution with ±2LSB accuracy), allowing us to obtain a high number of decimal places. During development, a balance must be found between information accuracy and usefulness. Hence, it should be noted that, although the measuring equipment is capable of operating with a higher resolution, the measured values in the range 0 gf to 99.9 gf are presented rounded to a single decimal place. In the hundreds range, from 100 gf to 500 gf, the measured values are rounded to the nearest unit. This procedure guarantees a balanced rounding and speeds up the comparison of the values obtained in the measurements with the values declared by the monofilament manufacturers. The same approach can be used to evaluate other monofilament calibers.

The PLDS is intended to perform high-precision movements. For this, a stepper motor, powered by a ULN2003A-based electronic driver, and a toothed belt drive are used. This setup is capable of making minimum increments of 0.08 mm in the displacement steps at the time of a given measurement. Still, regarding the characteristics of the linear displacement component, a total space of approximately 140 mm can be covered, enough to manipulate the various models of monofilaments available on the market. Table 3 shows the most relevant technical specifications of the proposed equipment. The measurement range, resolution, precision, and accuracy were obtained through the calibration process with the MecMesin Multitest machine.

When the equipment is operated in automatic mode, it is necessary to connect it to a computer through a USB Mini-B communication cable. This communication, through a specific computer application developed for this purpose, allows us to operate the equipment and receive all the information related to each monofilament evaluation. As for the energy source of this equipment, it is powered by an external source of 9VDC.

## 4. Methods, Results, and Discussion

Analyzing the literature reveals that there is still uncertainty about the effective accuracy and precision of monofilaments in their use to test the loss of sensitivity in the diabetic foot. Therefore, the methodology applied in the present study was organized with the aim of obtaining a concrete answer, performing a rigorous metrological verification of fourteen SWM of 10 gf (from different manufacturers) most used by the medical community in general. For this verification, equipment capable of simulating the conventional procedure used by healthcare professionals was developed (previously described). This procedure registers the behavior of the nylon filament until it deforms with the recommended curvature, at which point the force declared by its manufacturer will have been applied. The equipment will also be able to perform fatigue tests to assess performance after several repetitions.

In the following subsections, a set of different scenarios that were used to explore the proposed calibration equipment, as well as the analytical model of the monofilament, will be presented.

### 4.1. Equipment Validation

To evaluate the measurement performance of the proposed equipment, it was submitted to a series of validation procedures through controlled tests with a MecMesin compression machine. This machine, with a valid calibration certificate from a national metrological institution, is able to produce a controlled pre-determined force with a precision of ±0.01 N. The MecMesin machine can also provide information about the force that was produced with a resolution of ±0.0001 N (in other words, the actuation control system is not able to achieve the same resolution as the measuring system). The test protocol consisted of compressing the FSS of the proposed equipment into several measurement points plus a random increase of ≤25%, in rounds of thirty measurements for each. The mean compressive force values of each set and the respective standard deviation obtained by the MecMesin machine and the proposed equipment were recorded. The obtained results can be observed in Table 4. It should be noted that the standard deviation (SD) values indicated by the MecMesin machine were deliberately provoked to verify whether the FSS equipment followed the same behavior.

In addition, the obtained results were plotted using Bland–Altman plots [32]. This type of representation provides a valuable visual representation to observe the agreement between two measuring instruments. It allows for a quick assessment of comparability or discrepancies between the instruments. The plot displays the mean difference between the measurements obtained from the two instruments as a horizontal line, which, if close to zero, indicates little to no systematic bias. Moreover, the limits of agreement (corresponding to ±1.96 standard deviations, or a 95% confidence interval), represented by two horizontal dashed lines above and below the mean difference line, offer insight into the range within which most of the differences lie. Any deviation from a consistent distribution of points around the mean difference line may suggest proportional bias, wherein differences vary with measurement magnitude. Outliers in the plot are easily identifiable and can highlight potential measurement errors or instances where one instrument performs exceptionally well or poorly.

**Figure 6:**
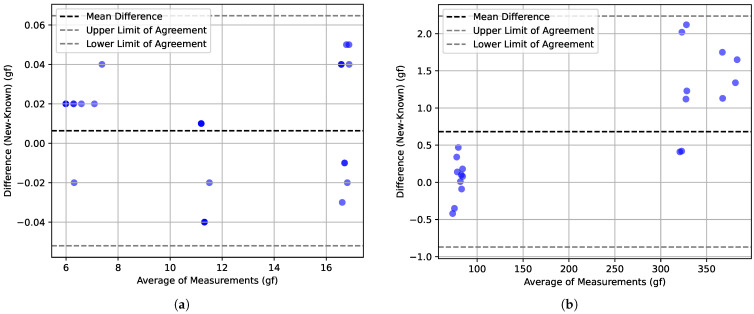
Bland–Altman plots comparing a reference device (MecMesin) and the proposed equipment (FSS): (**a**) for reference forces near the 10 gf monofilament operation range; (**b**) for reference forces near the operational range limit of the proposed equipment.

In Figure 6 the very good performance of the proposed equipment can be observed, either in the 10 gf monofilament operation range (Figure 6a) but also near its operational limits (Figure 6b). Near the 350 gf reference point, it can be observed that the values’ differences are higher and always positive. This is due to the combination of numerical rounding in the MecMesim system and also in the proposed system. This aspect does not have relevance for the specific purposes of this study, whose operational range is near 10 gf, for which excellent metrological properties were observed.

The obtained results showed that the FSS equipment presented very precise and consistent measurements, which is a positive indication of the performance of the proposed equipment. The comparison of the values obtained by the FSS equipment with the values measured by the MecMesin machine verified the reliability of the measurements carried out by the FSS. The very similar standard deviation values between the two devices indicate that the FSS measurements are consistent and demonstrate that the proposed equipment has a good repeatability capability in its measurements. It should be noted that the mean relative error, calculated on all tests of the various measurement ranges, was approximately 0.28%. These results also show that the FSS equipment is able to perform the SWM buckling force measurement tests with accurate and reliable results. If the analysis interval is the force range that is often used for diabetic foot-related procedures, the proposed system can achieve the highest measuring performance.

### 4.2. Monofilament Axial Compression

After ensuring the optimal accuracy and consistency of the calibration equipment, a set of tests was performed with monofilament devices used for the plantar sensitivity test. Fourteen monofilaments, with a force threshold of 10 gf, were carefully chosen from diverse manufacturers. All the devices were in new condition, and none had information besides the critical force. All tests were conducted at a stable room temperature of 20 °C to minimize environmental influences on the results. The monofilament, clamped in the PLDS and starting from a reference position, was longitudinally displaced towards the FSS, by 10 mm (necessary displacement to make the buckling curvature recommended), applying force in a perpendicular direction in relation to the platform. This setup mirrored real-life scenarios and facilitated precise plantar sensitivity evaluations. To assess the impact of different monofilament progression speeds on plantar sensitivity, two values were used: 4 mm $s^{-1}$ and 8 mm $s^{-1}$. Again, these speed choices were chosen with the purpose of mimicking the human-made procedure. It should be noted that the tests with different speeds were performed with a difference of 24 h between them to minimize potential fatigue or memory effects on the monofilament.

**Figure 7:**
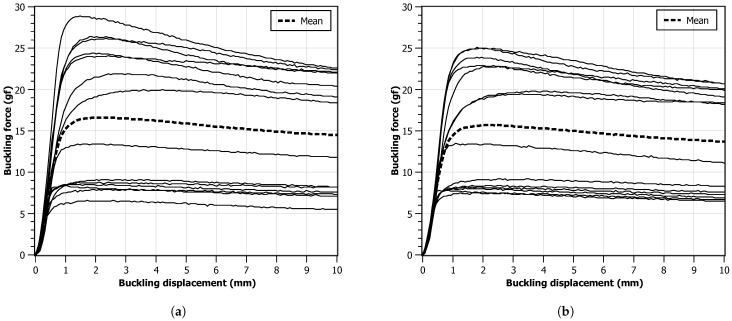
Force values measured with the FSS as a function of monofilament displacement. Each line represents one monofilament. The dashed line is calculated as the mean value of the other lines: (**a**) progression speed of 4 mm $s^{-1}$; (**b**) progression speed of 8 mm $s^{-1}$.

Figure 7 illustrates the force–displacement charts obtained from the experimental data. Upon compression initiation, two distinct regimes become apparent: a linear regime before reaching the critical force $F_{c}$, and a buckling regime that follows thereafter. The force contours of the tested devices can be categorized into two groups. The first group exhibits an incremental force rise up to its critical value, maintaining relative stability throughout the entire displacement range. In contrast, the second group demonstrates an overshooting phenomenon, with the critical force slowly decaying during buckling. This behavior may arise from the utilization of monofilament material, which offers superior resistance to compression compared to deflection. By comparing the two charts, it can be observed that, when the displacement speed is higher, this effect is less visible. Looking at the final values, it can be observed that all monofilaments provided a force that is different from the expected 10 gf, and many of them exerted a force twice the expected value. A smaller diversity of behaviors for the 8 mm $s^{-1}$ scenario can also be observed.

### 4.3. Monofilament Stress Tests

Utilizing a monofilament in isolation for testing purposes may not fully represent the real-world conditions in which the device is intended to be used. To better simulate actual usage scenarios, it was decided to investigate the behavior of each of the fourteen monofilaments through a sequence of 20 consecutive compression cycles, with a time interval of 3 s between consecutive compressions. The experiments were conducted at a room temperature of 20 °C and at two distinct displacement speeds, as in the previous test. Again, tests with different speeds were performed with a difference of 24 h between them to minimize possible effects of memory or fatigue on the monofilament.

**Figure 8:**
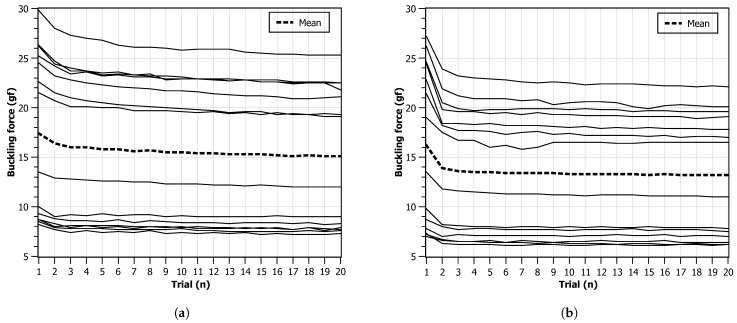
Critical force values during 20 trials. Each line represents one monofilament. The series mean value is calculated and represented as a dashed line: (**a**) progression speed of 4 mm $s^{-1}$; (**b**) progression speed of 8 mm $s^{-1}$.

Figure 8 presents charts for the critical force measurements obtained for each monofilament across the 20 cycles. It can be observed that all monofilaments have a higher critical force in the first compression trial that starts to decay with repeated compressions. On average, this effect is slower and smaller when a lower displacement speed is used. For the higher displacement speed, most monofilaments reach a force plateau after the second compression. It can also be observed that, in both scenarios, the average value is above the expected 10 gf, being around 15 gf for the 4 mm $s^{-1}$ and around 13 gf for the 8 mm $s^{-1}$ case. In the faster displacement scenario, it can be observed that two monofilaments do not provide a contour pattern compatible with all the others. This may point to geometrical defects during manufacturing or non-homogeneous material distribution.

**Figure 9:**
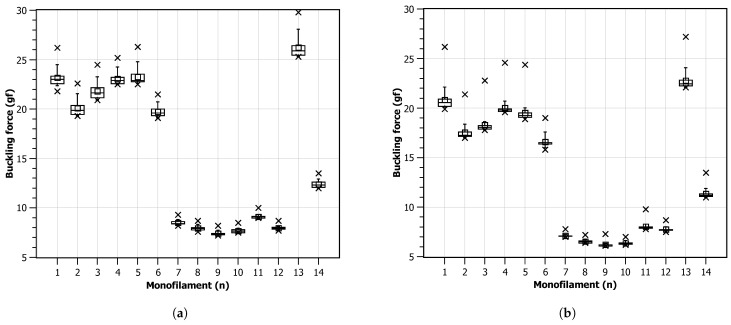
Critical force values after 20 trials: (**a**) progression speed of 4 mm $s^{-1}$; (**b**) progression speed of 8 mm $s^{-1}$.

Figure 9 shows the charts for the critical force values for each monofilament independently. Two groups (1 to 6 and 7 to 12), showing to share similar behavior during the experiments, can clearly be observed. In general, due to the observed repeatability, it was found that the monofilament device can be reliable for repeated usage but a stable high-precision manufacturing process is necessary.

The above-presented experiments and the reported results provide valuable insights into the monofilament’s performance under repeated compression, reflecting its ability to withstand continuous usage and offering a more comprehensive understanding of its behavior in practical applications, since these instruments, after disinfection, can be used several times a day in several patients.

### 4.4. Theoretical Value of Critical Force

By substituting the measured values of a 10gf monofilament sample

d=0.61mm=0.00061m

into Equation (4), we can compute

$$I=\frac{\pi \cdot {\left(0.00061\right)}^{4}}{64}$$

To calculate the effective length ($L_{\mathrm{effective}}$), the boundary conditions must be considered. For a clamped-free condition, the effective length is considered to be 0.7 times the actual length. Therefore,

L=38mm=0.038m

$L_{\mathrm{effective}}=0.7\times 0.038=0.02660\;m$

Now, the Young’s modulus (*E*) of the nylon material must be defined. For the purpose of this example, a typical value for nylon was assumed, being approximately 2 GPa (2 $\times \;10^{9}$ Pa). All the values in Euler’s buckling equation can be replaced (3):

$$F_{\mathrm{critical}}=\frac{\pi^{2}\cdot \left(2\times 10^{9}\right)\cdot \left(\frac{\pi \cdot 0.00061^{4}}{64}\right)}{0.02660^{2}}$$

Simplifying the equation, the following is obtained:

$$F_{\mathrm{critical}}\approx \pi^{2}\cdot \left(2\times 10^{9}\right)\cdot \left(\frac{\pi \cdot 0.00061^{4}}{64}\right)\cdot \left(\frac{1}{0.02660^{2}}\right)\approx 0.18961\;N$$

The resulting value will be in units of force, such as newtons (N). To make a direct comparison with the target values of the monofilaments possible, it should be converted to the mass unit as follows:

$$m=\frac{F}{a}=\frac{0.18961}{9.80665}=0.01933\;\mathrm{kg}=19.33\;g$$

Therefore, this monofilament has a critical buckling force of approximately 19.33 gf, well above the 10 gf declared by the manufacturer but compatible with some of the obtained test results, as shown in Figure 7. However, it should be noted that these calculations assume linear elastic behavior and neglect any other factors that may affect the buckling behavior, such as imperfections or other deformation complexities. Additionally, the actual buckling behavior of a nylon filament may also depend on other factors like temperature, moisture, and manufacturing tolerances, as previously mentioned.

### 4.5. Material and Geometry Variations

Using the measures obtained during the monofilaments tests (in Section 4.2), the material’s Young’s modulus *E* was estimated for each device, as seen in Table 5. Considering all tested devices, the average value for Young’s modulus is 2.74 GPa, which is within the expected value range for a nylon polymer. These values were then used to calculate how the monofilament behaves when there is slippage of the free extremity, as shown in the rightmost column. The effective length $L_{e}$ of the monofilament is a quadratic parameter in Equation (3), showing that inadequate friction conditions between the monofilament and the skin surface will have a very significant impact on the result and quality of the SWM test.

From Equation (3) it can be observed that, while Young’s modulus appears as a linear coefficient, the (monofilament’s) length is squared, being more relevant for the force’s final value. In Equation (4), a linear factor in Equation (3), it can be observed that *d* has a fourth-order exponent, causing small variations in the variable to have a high impact on the results.

**Figure 10:**
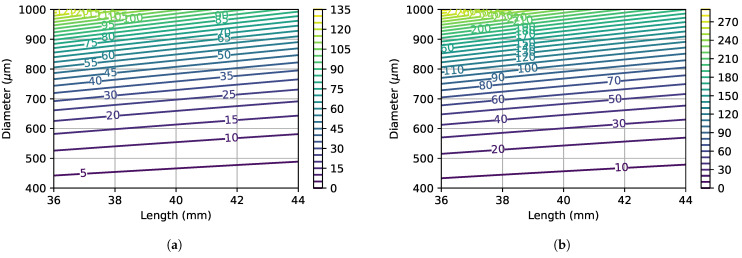
Critical force as a function of the monofilament length *L* and diameter *d*. Values are in gf. Young’s moduli correspond to the minimum and maximum values that were estimated during calibration tests. (**a**) *E* = 1.68 GPa. (**b**) *E* = 3.65 GPa.

Figure 10 shows the critical force as a function of monofilament length and diameter, for two distinct Young’s moduli. The effect of increased length and reduced diameter (for example considering a 10% manufacturing tolerance) can lead to a severe difference in the critical force. By observing the contour lines’ orientation, the importance of diameter variations can be identified as highly relevant to the devices’ performance (as expressed in Equation (4)). The correct choice of material is also an important factor to consider for manufacturing purposes, as a lower Young’s modulus value allows for a higher dimensional tolerance.

Deflection due to the monofilament weight can have a major impact on the device performance since, as seen in Figure 7, there are two distinct regimes during compression, mediated by the occurrence of the critical buckling force. When the monofilament has a higher length and smaller diameter, assuming equal material properties, then the deflection will be higher, and the transition between the two observed regimes does not present an overshoot.

In the center of Table 6 (in bold typeface), the deflection value δ is shown, due to its own weight, for the longest (*L* = 41 mm) and thinnest (*d* = 0.46 mm) monofilament of the experimental test set. For this case, the relative deflection in relation to its diameter δ(%d) was 17.8%. This monofilament (and others in the test set with similar properties) shows no overshoot in the force–displacement contours of Figure 7 (the monofilament group whose curves are shown lower in the chart). For the shortest (*L* = 38 mm) and thickest (*d* = 0.71 mm) monofilament (not shown in Table 6), the deflection was δ = 55 μm, corresponding to 7.8% of its diameter.

From Equations (4) and (7), it can be seen that both diameter *d* and length *L* have a high impact on the monofilament deflection behavior, as can be observed in the variations presented in Table 6. The highest relative deflection δ(%) was obtained for a 10% increase in the monofilament length and a 10% decrease in its diameter, leading to a deflection increase of 81%.

## 5. Conclusions

In this article, a novel equipment designed for the automatic metrological evaluation of SW monofilaments was introduced, providing a valuable tool for both objective assessment and training of health professionals in the SWM test procedure. The present study demonstrates the system’s capability to automatically evaluate monofilaments, yielding detailed and precise displacement vs. force contours that characterize the behavior of each individual device.

### 5.1. Key Contributions

While some studies have consistently recommended the use of monofilament as a fast and cost-effective tool for assessing diabetic neuropathy [3], concerns have been raised about the reliability of the measurements obtained with this device, contradictorily not encouraging its usage [10]. The current paper introduces a solution to resolve this conflict, eliminating the main limiting factor in the use of monofilament. The proposed equipment allows users to benefit from the advantages of the monofilament while enhancing confidence in its usage, thereby supporting more accurate clinical decisions and improving healthcare outcomes.

Notably, the presented findings reveal that the forces applied by the monofilaments may deviate from the manufacturers’ specifications, highlighting the importance of accurate calibration and testing parameters for reliable clinical assessments. Understanding these discrepancies contributes to the refinement of neurological diagnosis and rehabilitation strategies, enabling more effective treatment decisions.

In addition to presenting a new device for the automatic metrological evaluation of SW monofilaments, this study encompasses the development of analytical models to comprehend the influence of each variable on the critical buckling force during monofilament compression. These analytical models serve as valuable tools to investigate and dissect the complex behavior of monofilaments under compression, allowing us to gain deeper insights into the factors that significantly impact the critical buckling force. By employing these analytical models, it was possible to discern the interplay between various parameters, shedding light on their individual contributions to the overall behavior of the monofilaments during the compression process.

Using this calibration equipment and the analytical model, various tests were conducted on a range of commercially available monofilament devices. The combination of experimental results and analytical modeling provides deeper insights into their operational characteristics and allows us to estimate the potential errors during its use. With this knowledge, healthcare professionals can make more informed decisions when interpreting results and devising adequate follow-up routines and treatment plans for patients based on accurate and precise data.

### 5.2. Challenges and Constraints

The primary objective of the proposed device is to offer regular calibration of the monofilament within a clinical setting, ensuring consistent accuracy in patient assessments. The prototype presented here was meticulously designed to be both robust and user-friendly. However, to fully validate its effectiveness, it is crucial to gather usage data from a diverse pool of users. This step, which remains to be undertaken, will provide valuable insights for refining the device. User feedback, collected during routine clinical use, will be instrumental in guiding further enhancements to both the hardware and software, allowing the authors to optimize the device’s performance and usability.

The integration of the proposed device into a clinical setting requires the development and implementation of new procedures as part of routine operations. Key aspects such as the timing and frequency of calibrations, as well as the assignment of responsibilities, must be clearly defined. It is also crucial to outline the implications of the calibration results, considering all variables that could impact the device’s performance. Furthermore, comprehensive training sessions will be necessary, and it is essential to address the expectations and concerns of all stakeholders. These aspects present significant challenges that must be carefully managed, and only by addressing them can the objectives that motivated the development of the proposed device be fully achieved.

Regarding the materials used in the construction of the monofilament, while a broader range of materials could have been explored, our focus was on those most commonly cited in the literature. This choice was made to align with established clinical practices and ensure the device’s immediate applicability in real-world settings.

### 5.3. Future Directions

The equipment is planned to be implemented within a real clinical practice setting, specifically in a service dedicated to diabetic foot monitoring. It is important to evaluate the level of error associated with ongoing assessments, taking into account the operational practices of the service and the procedures for utilizing monofilaments. Understanding these error margins will be crucial in improving the accuracy of diabetic foot assessments. Additionally, it is important to evaluate the user experience with the software that was developed for operating the proposed equipment. Only an easy-to-use system can ensure the adequate integration of the system, thus achieving its purpose.

Furthermore, efforts are being made to develop comprehensive training initiatives for healthcare professionals who administer the Semmes–Weinstein test. The goal is to heighten their awareness and sensitivity to significant variations in the force applied by the monofilaments during testing. According to the literature [33,34], the tactile sensitivity of an operator when applying force is approximately 10 gf, which is comparable to the force required for the monofilament test. However, there is potential to enhance this sensitivity further by employing neurofeedback techniques, which could improve the responsiveness of skin mechanoreceptors [35]. This training can be essential in identifying defective devices and improving diagnostic consistency.

### 5.4. Study Impact and Outlook

In summary, this study not only advances the reliability and practical application of monofilament-based diabetic neuropathy assessments but raises awareness for the continued improvement in clinical tools. The proposed calibration device and analytical model enhances the knowledge and precision of current methodologies. Furthermore, it promotes a more robust and precise usage of SW monofilaments, ultimately contributing to improvement in clinical practices and decision-making processes.

## Figures and Tables

**Table 1:** Summary of factors that impact the operation characteristics of monofilament.

Material	Geometry	Loading Conditions	Environment
Young’s Modulus	Length and diameter	Compressive load	Temperature
Yield stress	End conditions	Load distribution	Moisture
Poisson’s ratio	Imperfections	Loading rate	

**Table 2:** Technical specifications of the compression testing machine—MecMesin Multitest 10-i.

Specification	Value/Description
Load cell ref.	ILC 10 N
Range (N)	0 to 10
Resolution (N)	>0.0001 (1:6500)
Precision (N)	±0.01 (0.1% fs)
Acquisition rate (Hz)	10
Displacement velocity (mm $s^{-1}$)	0.08 (**a**)

(**a**) Velocity can be controlled.

**Table 3:** Detailed equipment specifications.

Specification	Value/Description
Range (gf)	(**a**) 0.1 to 99.9 and (**b**) 100 to 500
Resolution (gf)	(**a**) 0.1 and (**b**) 1
Precision (gf)	(**a**) ±0.03 and (**b**) ±0.25
Accuracy (%)	(**a**) ±0.36 and (**b**) ±0.68
Displacement resolution (mm)	0.08
Power supply (VDC)	9
Computer interface	USB mini-B connector; Specific Windows app
Modes	Linear by limit switch and by number of steps

(**a**) Rounds the measurement value to a single decimal place; (**b**) rounds the measurement value to unity.

**Table 4:** Mean force and related standard deviation (±SD) from the MecMesin and FSS equipment. The MecMesin row shows the mean and standard deviation the generated forces, and the FSS row shows the measured forces’ related statistics. For each reference point, a series of thirty measures were obtained at a room temperature of 20 ºC.

Reference Point	6 gf	10 gf	15 gf	20 gf	50 gf	300 gf
MecMesin	6.4 ±0.5	11.3 ±0.1	16.7 ±0.1	22.2 ±0.6	80.0 ±3.7	344.3 ±26.2
FSS	6.4 ±0.5	11.3 ±0.1	16.7 ±0.1	22.3 ±0.6	80.0 ±3.9	345.6 ±26.3

**Table 5:** Monofilament properties for four distinct devices. The materials’ Young’s moduli *E* was estimated from previously obtained geometrical dimensions (diameter *d*, length *L*, and critical force measures $F_{c}$, considering clamped–pinned boundary conditions k=0.7, corresponding to the device calibration procedure setup conditions). Using *E*, a new critical force was calculated considering clamped–sliding boundary conditions k=2.0. (First column represents a number attributed to a given monofilament device for reference purposes.)

#	*d*	*L*	*E*	*I*	$F_{c}$ (k = 0.7)	$F_{c}$ (k = 2.0)
	**(mm)**	**(mm)**	**(GPa)**	**($m^{4}$)**	**(gf)**	**(gf)**
1	0.61	38	2.99	6.797 × 10^−15^	28.91	3.54
2	0.46	41	3.65	2.198 × 10^−15^	9.80	1.20
3	0.71	38	1.68	12.474 × 10^−15^	29.81	3.65
4	0.52	38	2.65	3.589 × 10^−15^	13.50	1.65

**Table 6:** Deflection of the devices’ free extremity due to their own weight, considering 10% variations (increase or decrease) in the monofilament length *L* and diameter *d*. Absolute values δ are in μm. Relative deflection δ(%) values have the 10 gf monofilament deflection (center, bold typeface) as reference. Relative deflection δ(%d) values have the diameter of each monofilament as reference. The reference monofilament has length *L* = 41 mm, diameter *d* = 0.46 mm, Young’s modulus *E* = 3.65 GPa, and density 1100 kg $m^{-3}$.

d∖L	L(−10%)			L(0%)			L(+10%)		
	δ(μm)	δ (%)	δ(%d)	δ(μm)	δ (%)	δ(%d)	δ(μm)	δ (%)	δ(%d)
d(−10%)	66.26	−19%	16.0%	100.99	23%	24.4%	147.87	81%	35.7%
d(0%)	53.67	−34%	11.7%	**81.81**	**0%**	**17.8%**	119.77	46%	26.0%
d(+10%)	44.36	−46%	8.8%	67.61	−17%	13.4%	98.98	21%	19.6%

## Data Availability

Data are available upon request to the corresponding author.
